# Sinonasal Extramedullary Plasmacytoma With Rare Osteolytic Lesions

**DOI:** 10.7759/cureus.14220

**Published:** 2021-03-31

**Authors:** Georgia Pantazidou, Ioannis Papaioannou, Eleni Karagkouni, Ioannis Fragkakis, Panagiotis Korovessis

**Affiliations:** 1 Otolaryngology - Head and Neck Surgery, General Hospital of Patras, Patras, GRC; 2 Orthopedics, General Hospital of Patras, Patras, GRC

**Keywords:** nasal cavity, extramedullary, plasmacytoma, osteolytic lesions, extramedullary plasmacytoma

## Abstract

Plasmacytomas are malignant tumors characterized by abnormal monoclonal proliferation of plasma cells. They originate either from bone or soft tissue and could be primary or a part of a systemic process during the course of multiple myeloma. Extramedullary plasmacytomas (EPs) in the sinonasal tract or nasopharynx are rare and mostly presented as case reports. We describe a unique case of multiple myeloma involving the nasal cavity and the paranasal sinuses with osteolytic expansile lesions of the first cervical vertebrae (atlas), the clavicle, and the skull in a 51-year-old man. The diagnostic approach was challenging, and finally the biopsy of the extramedullary tumor settled the diagnosis of multiple myeloma. The patient underwent posterior occipitocervical fusion due to upper cervical spine instability due to atlas osteolysis. The patient had an uneventful recovery, and he was finally referred to the hematology department. EP of the nasal cavity is a rare entity and requires a high index of suspicion. EP should be included in the differential diagnoses of nasal cavity masses, especially in males in the age group of 50-60 years. A thorough clinical history, examination, and proper laboratory and radiological investigations are important to settle an accurate diagnosis to initiate treatment as soon as possible. Timely diagnosis ensures a better prognosis and deters the progression of the disease.

## Introduction

Plasmacytomas are malignant tumors characterized by abnormal monoclonal proliferation of plasma cells. They originate either from bone or soft tissue (extramedullary plasmacytoma [EP]) [1]. EPs could be primary (without evidence of disease in other foci) and rarely can turn to multiple myeloma (MM) as part of a systemic process during the course of MM. According to International Myeloma Working Group (IMWG), the diagnostic criteria to settle the MM diagnosis are as follows: clonal plasma cells in bone marrow more than 10% or biopsy-confirmed bone plasmacytoma (BP) or an extramedullary manifestation accompanied by either (1) Calcium elevation, Renal dysfunction, Anaemia and Bone disease (CRAB) criteria (hypercalcemia, renal dysfunction, anemia, more than one bone lesions) or (2) clonal plasma cells in bone marrow more than 60%, ratio of involved/uninvolved free light chains more than 100, and at least one focal lesion more than 5 mm [1]. The etiology of EPs is unknown, while the first case of EP was reported in 1905 by Schridde [2]. EP affects men three to four times more often than women and typically occurs in the sixth to seventh decade, with more than 95% of cases occurring in patients above 40 years of age [3]. EPs are very rare tumors, although the sinonasal tract or nasopharynx is the main affected area. They represent less than 1% of all head and neck malignancies, and the reports in the current literature concern only case presentations [4]. Nasal EPs usually manifest as nasal obstruction, epistaxis, progressive dyspnea, and local pain. The rarity of this tumor accompanied by longstanding non-diagnostic clinical manifestations represents a diagnostic and therapeutic challenge for any ear, nose, and throat (ENT) surgeon. We describe a unique case of MM involving the nasal cavity and the paranasal sinuses with osteolytic expansile lesions of the first cervical vertebrae (atlas), the clavicle, and the skull in a 51-year-old man who experienced diplopia in the left eye, worsening headache, and neurologic symptoms due to the dynamic instability of the upper cervical spine. Our case is also unique because the diagnosis of the disease would not be possible if no biopsy has been taken from the nasal cavity, as the other lesions were inaccessible for biopsy.

## Case presentation

A 51-year-old man presented to our emergency department with photophobia of the left eye, dizziness, cervical stiffness, and pain. His clinical manifestations began one month ago with worsening headache and diplopia in the left eye. Our patient mentioned severe nasal obstruction for at least one year before admission, although he did not receive any specific medical consultation or any treatment. Further evaluation of the medical history did not reveal purulent rhinorrhea, epistaxis, or facial nerve palsy. Our patient did not have major comorbidities as he mentioned only idiopathic hypertension treated with irbesartan.

**Figure 1:**
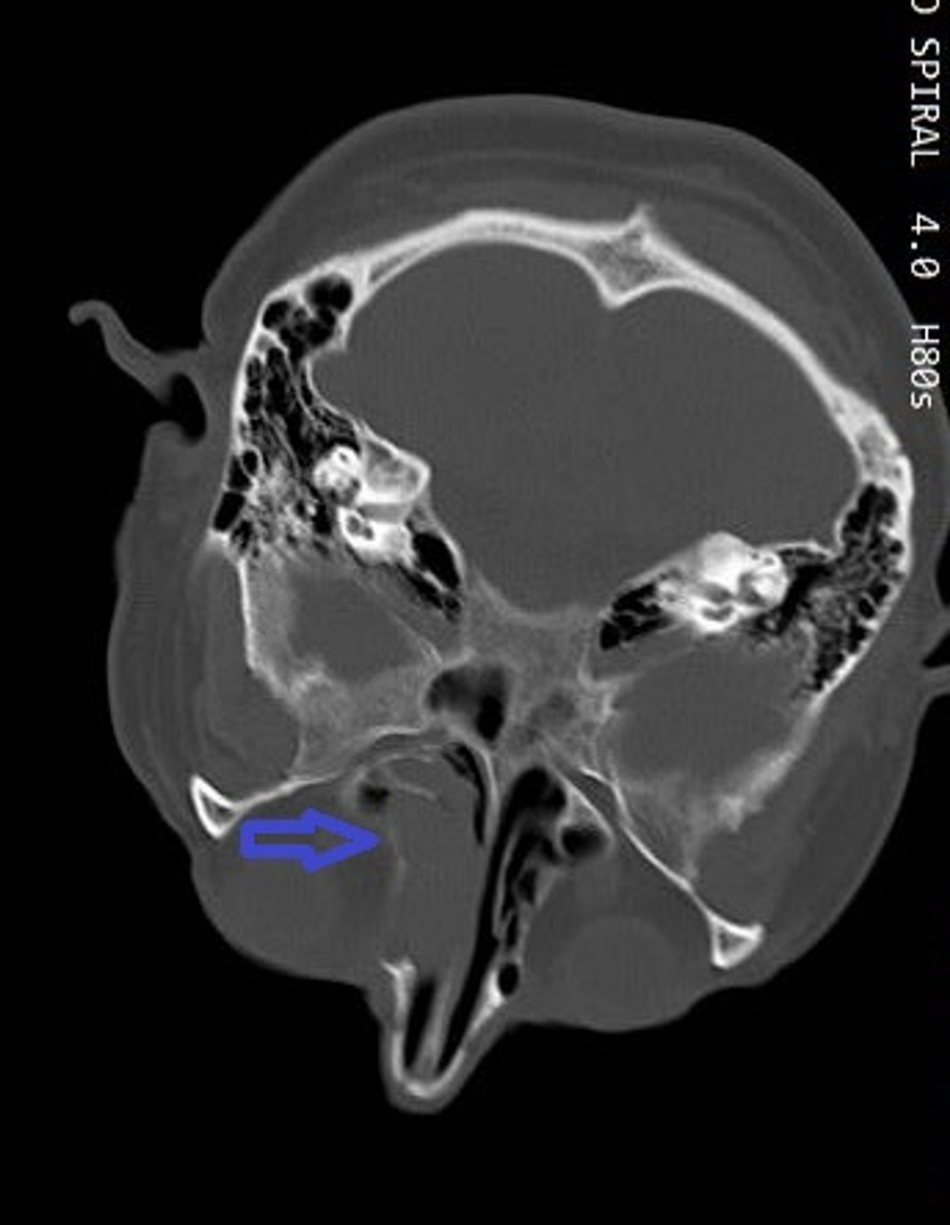
A CT scan (axial view) showing a heterogenous soft tissue mass filling the left nasal cavity (blue arrow) obstructing the choana.

**Figure 2:**
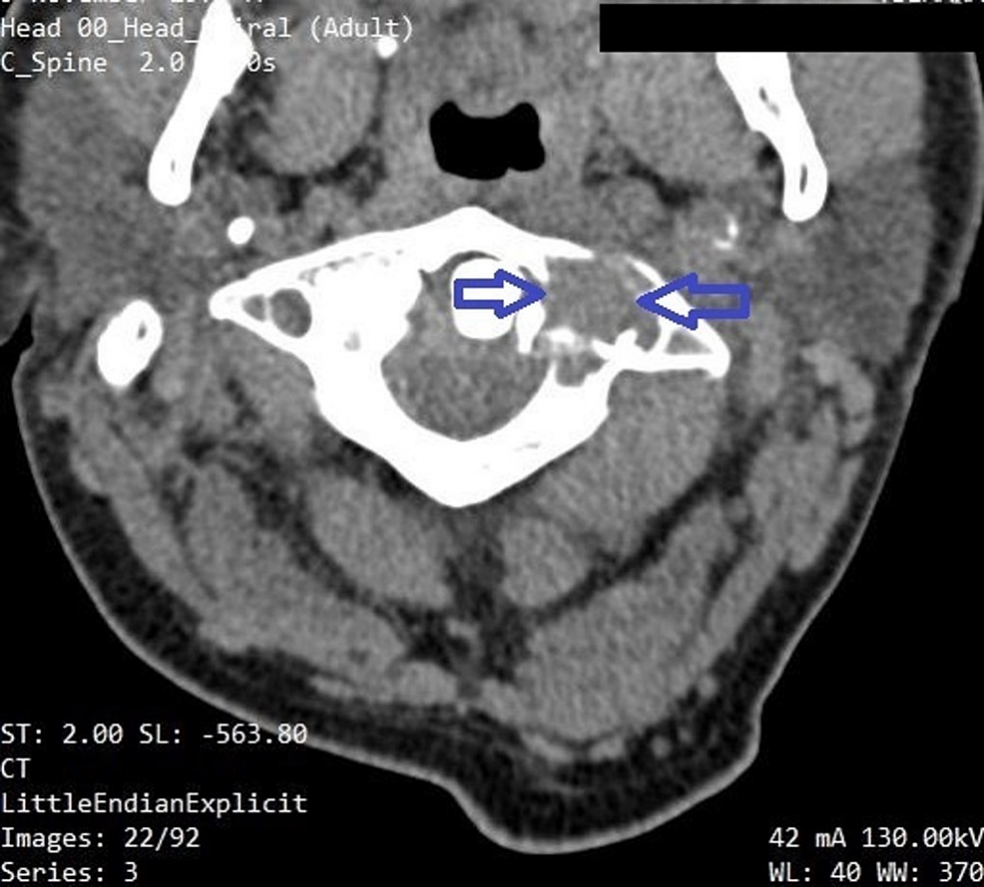
A CT scan showing an extensive osteolytic damage to the atlas (blue arrows).

**Figure 3:**
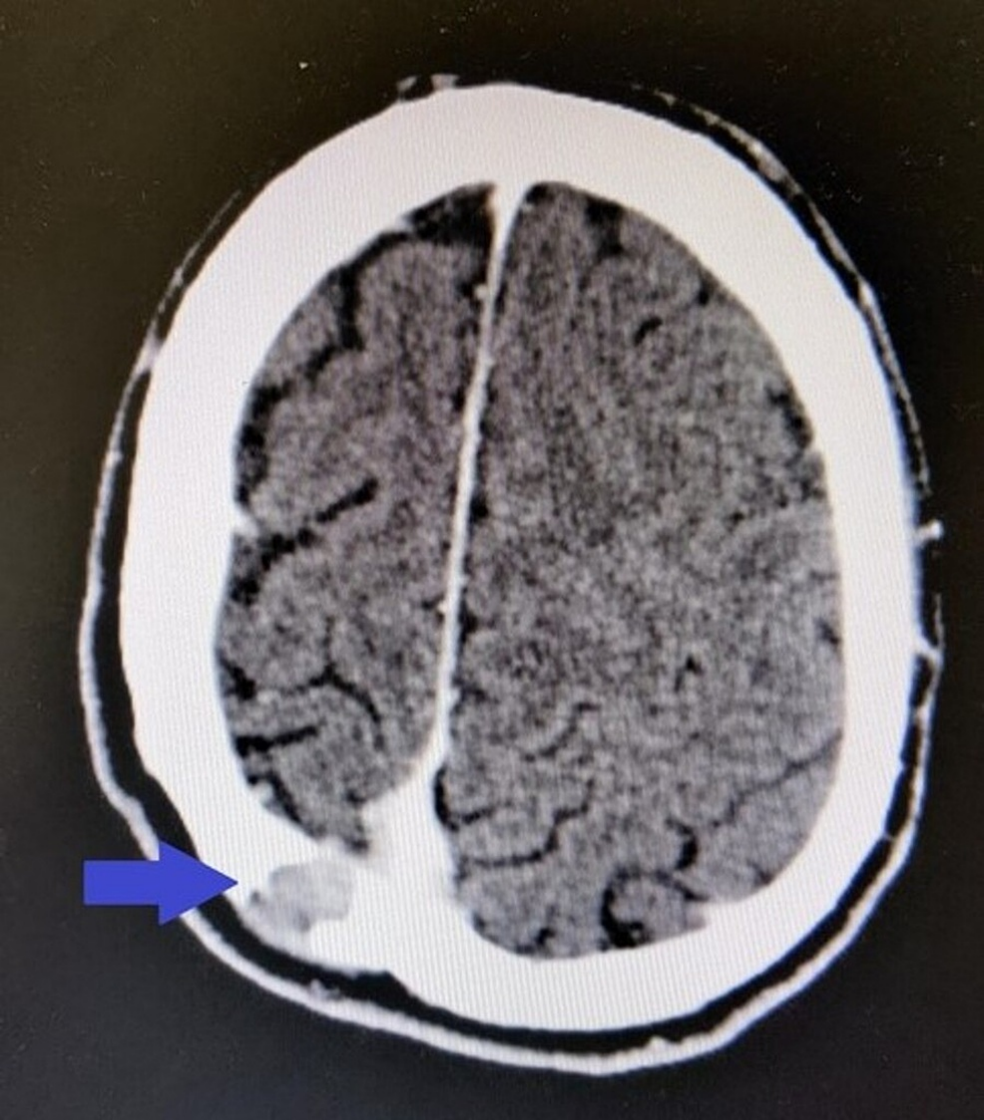
A CT scan showing an osteolytic damage to the skull occipitally (blue arrow).

**Figure 4:**
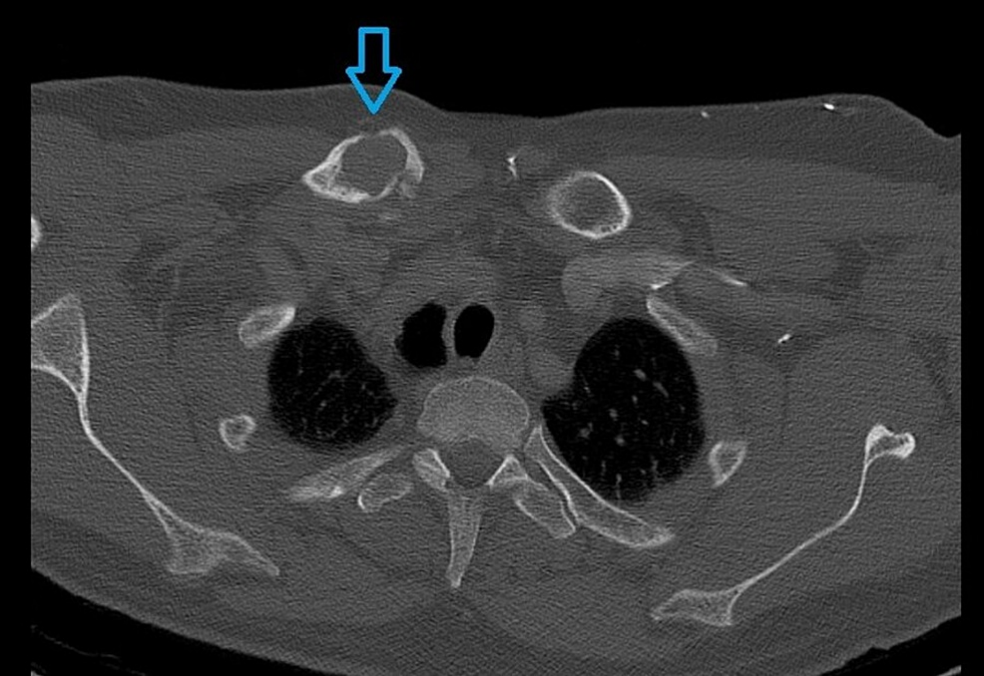
A CT scan showing an osteolytic damage to the clavicle (blue arrow).

Endoscopic examination of the nasal cavity was impossible due to cervical instability. Lumbar puncture was performed, without significant findings. Routine blood examinations were within normal limits (white blood cells: 7.3 × 10^9^/L; hemoglobin: 14.6 g/dl; erythrocyte sedimentation rate: 12 mm/h; C-reactive protein: 2 mg/L; calcium: 9.3 mg/dL; creatinine: 0.9 mg/dL; urea: 15 mg/dL). Computed tomography (CT) of the nasal cavities and paranasal sinuses (axial view) with intravenous contrast showed an heterogenous soft tissue mass filling the left nasal cavity with signs of mass effect of the medial wall of the left maxillary sinus, locally thinned with no signs of erosion (Figure 1). CT scan evaluation revealed an extensive osteolytic damage to the left lateral mass of the atlas (Figure 2), a minor osteolysis to the occipital aspect of the skull (Figure 3), and one more lesion to the sternal aspect of the right clavicle (Figure 4).

**Figure 5:**
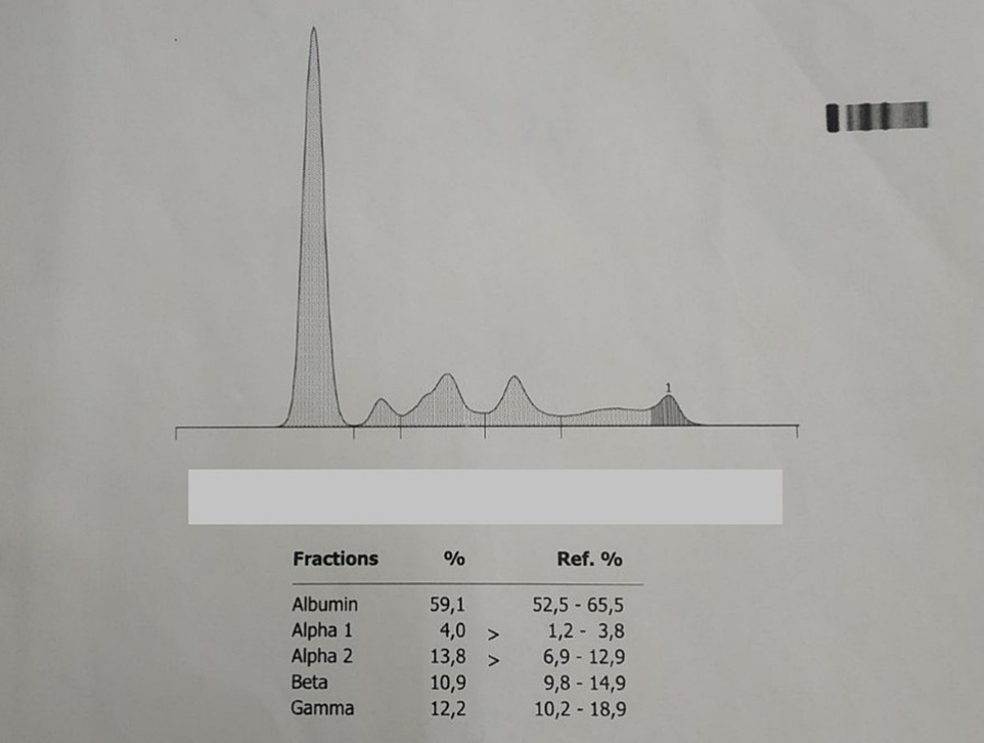
Diagram of serum protein electrophoresis.

**Figure 6:**
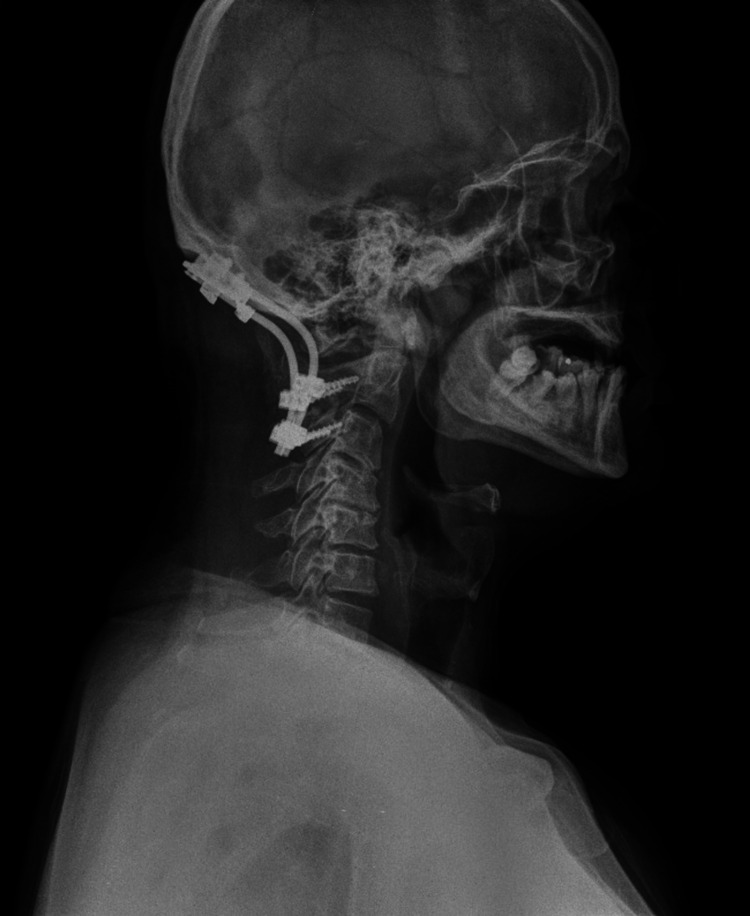
Profile X-ray of the cervical spine after occipitocervical fusion.

Lung X-ray did not show the presence of secondary deposits, and the blood tests were within normal limits. Serum protein electrophoresis (Figure 5) was normal, and Bence Jones protein was absent from our patient’s urine tests. Fine needle aspiration biopsy from the clavicle would be a reasonable diagnostic approach, although we had scheduled posterior occipitocervical fusion accompanied by endoscopic biopsy of the nasal tumor. Unfortunately, our department is not equipped with positron emission tomography (PET) scan to diagnose possible more accessible lesions. The patient underwent occipitocervical fusion via posterior approach for stabilization of the cervical spine (Figure 6).

The left lateral mass was completely eroded by the mass with disruption of the anterior arch and erosion of the transverse ligament. This sequalae led to upper cervical spine instability accompanied by significant neck pain and severe restriction of neck movements. In the same anesthesia and after stabilization of the upper cervical spine, we performed biopsy of the nasal tumor endoscopically. Immunohistochemical staining showed positive expressions of CD79a, CD138, CD56, 30% Ki-67, and vimentin in tumor cells, while negative expressions of other tumor markers. The biopsy from the atlas was not diagnostic. Based on these characteristics, the pathologist diagnosed the patient with plasmacytoma involving the nasal cavity and the paranasal sinuses. According to IMWG, our patient fulfilled the following criteria to settle the MM diagnosis: clonal plasma cells in an extramedullary manifestation accompanied by at least one focal lesion more than 5 mm.

The patient was then referred to the hematology department of our hospital for further treatment (radiotherapy and chemotherapy of the MM).

## Discussion

EP is a solitary lesion without clinical, histological, or radiological evidence of MM. It is divided into two entities depending on the origination of either bone or soft tissue [5]. The incidence of bone solitary EP is approximately 40% higher than that of soft tissue EP [6]. Solitary soft tissue plasmacytomas often remain localized and confer better prognosis compared to solitary BP. It may be isolated or the first manifestation of MM [7]. Τhe most common location for soft tissue EM is the head and neck (approximately 90%), especially the upper respiratory system (nasal cavities, sinuses, nasopharynx, and larynx), followed by the gastrointestinal system [8,9]. The median age at diagnosis of solitary EP is 55-60 years and is significantly lower in MM patients, while the male-to-female ratio varies from 1.2:1 to 2:1. [10].

Ιn our case, the patient had nasal obstruction for at least one year before admission and finally the diagnosis of MM was settled. This fact confirms the current evidence, which supports that EP is a precursor of MM. Literature supports that 11% to 30% of EPs can progress to MM in a period of 10 years [7]. The presence of EP should alert physicians for possible simultaneous osteolytic lesions. Imaging and laboratory testing are mandatory to confirm or exclude the diagnosis of MM. The exact etiology of EP is unknown, although it has been proposed that chronic stimulation of the mucosa of the upper respiratory tract by inhaled irritants or viral infection have been previously implicated [11]. To the best of our knowledge, this is the first report in the current literature of EP involving the nose and paranasal sinus regions and osteolytic expansile lesions of cervical vertebrae (atlas), the clavicle, and the skull at the time of diagnosis. The rarity of our case is based also on the fact that diagnosis of the disease would not be possible if no biopsy has been taken from the nasal cavity.

## Conclusions

We presented a rare case of EP of the sinonasal cavity with simultaneous osteolysis lesions in the cervical spine, skull, and clavicle in a male who had nasal obstruction for more than a year. The rarity of the case is based on the simultaneous presence of extramedullary manifestation accompanied by three bone lesions and due to the fact that the diagnosis was settled from the extramedullary nasal tumor biopsy. It is worth noting that EPs can be enlarged and progress to MM without any pathognomonic symptoms, which can alert patients to seek medical consultation. EP of the nasal cavity is a rare entity and requires a high index of suspicion. EP should be included in the differential diagnoses of nasal cavity masses, especially in males in the age group of 50-60 years. Physicians should have a high index of suspicion to distinguish EP from normal lymphoid tissue. Underestimation of such lesions could easily lead to misdiagnosis. A thorough clinical history, examination, and proper laboratory and radiological investigations are important to settle an accurate diagnosis to initiate treatment as soon as possible. Timely diagnosis ensures a better prognosis and deters the progression of the disease.
